# Opportunities and Challenges Surrounding the Use of Data From Wearable Sensor Devices in Health Care: Qualitative Interview Study

**DOI:** 10.2196/19542

**Published:** 2020-10-22

**Authors:** Ijeoma Azodo, Robin Williams, Aziz Sheikh, Kathrin Cresswell

**Affiliations:** 1 Usher Institute University of Edinburgh Edinburgh United Kingdom; 2 Institute for the Study of Science, Technology and Innovation University of Edinburgh Edinburgh United Kingdom

**Keywords:** wearable sensor devices, health care, data, qualitative

## Abstract

**Background:**

Wearable sensors connected via networked devices have the potential to generate data that may help to automate processes of care, engage patients, and increase health care efficiency. The evidence of effectiveness of such technologies is, however, nascent and little is known about unintended consequences.

**Objective:**

Our objective was to explore the opportunities and challenges surrounding the use of data from wearable sensor devices in health care.

**Methods:**

We conducted a qualitative, theoretically informed, interview-based study to purposefully sample international experts in health care, technology, business, innovation, and social sciences, drawing on sociotechnical systems theory. We used in-depth interviews to capture perspectives on development, design, and use of data from wearable sensor devices in health care, and employed thematic analysis of interview transcripts with NVivo to facilitate coding.

**Results:**

We interviewed 16 experts. Although the use of data from wearable sensor devices in health and care has significant potential in improving patient engagement, there are a number of issues that stakeholders need to negotiate to realize these benefits. These issues include the current gap between data created and meaningful interpretation in health and care contexts, integration of data into health care professional decision making, negotiation of blurring lines between consumer and medical care, and pervasive monitoring of health across previously disconnected contexts.

**Conclusions:**

Stakeholders need to actively negotiate existing challenges to realize the integration of data from wearable sensor devices into electronic health records. Viewing wearables as active parts of a connected digital health and care infrastructure, in which various business, personal, professional, and health system interests align, may help to achieve this.

## Introduction

Many countries now see digital transformation and digitally enabled self-management as a way to tackle key challenges of delivering health and well-being with limited health care resources to an increasingly aging population [1]. The transformatory potential of wearable devices forms part of a pervasive set of technological innovations characterized as Internet of Things (IoT) technology; these devices are typically mounted on wireless broadband technologies (ie, mediated by smartphones or equivalent devices) and are often linked to social media platforms. IoT refers to interconnected physical objects, devices, and systems that electronically communicate, receive, process, and transfer digital data with limited direct human input [2-5]. In health care practices, IoT connectivity allows the sharing of data from wearable sensor devices across various contexts, potentially encompassing the user, caregivers, clinicians, and the health record. Wearable sensor devices include tracking devices with capabilities to count steps, measure activity, and check heart rate. Advanced smartwatches have sensing capabilities that include temperature, blood pressure, stress, sleep quality, respiratory rate, physical activity, electrocardiograms, acceleration, and oximetry [6]. These capabilities are also present in wearable sensor devices used in hospitals and clinics, albeit with less investment in the device aesthetics. Technologies are considered “smart” if they monitor, collect, and send data. They differ from mobile devices such as smartphones in that they are wearable and do not require active data input or curation from the user.

The promise of IoT in health care lies in its potential to realize business benefits while providing a valuable, integrated health and social care service and superior experience for citizens and patients [7]. Growing internet connectivity and the increasing availability of smartphones and multiple portable devices facilitate digital information sharing, connected devices, and the possibilities for digital health care [8]. These interrelationships are part of a societal reorientation of new networked organizations that use information and digital data to allow new insights for business, populations, and health care delivery [9]. Here, wearable and sensor devices enable health and care practices through internet connectivity combined with mobile, miniature, pervasive computing [10,11]. It is hoped that emerging technologies will achieve a similar return on investment, productivity, and efficiency savings as seen in the financial and manufacturing industries [12,13]. Existing empirical studies of wearables in health care demonstrate the feasibility of creating technologies [14] and running data analytics [15,16]; small pilot studies have demonstrated the use of wearables in managing specific chronic conditions, including diabetes and high blood pressure, as well as contributing to data analytics [17-20]. There is also some evidence from trials that wearables can promote healthy behavior [21-23]. However, because these technologies have transformative potential, and there is limited adoption experience, their ultimate outcomes and significance cannot reliably be assessed at this moment. Nevertheless, the area is full of potential, with many apps already becoming routinely integrated with everyday life, with 1 in 5 Americans now possessing a smartwatch or fitness tracker [24].

There are a number of challenges associated with the use of wearable and sensor devices. Firstly, they are often conceptualized as consumer goods, which potentially limits their use as health apps. Secondly, they can change health care delivery models and also impact on the collection and dissemination of sensitive personal data. For example, continuous monitoring data collected through IoT technologies has the potential to improve the knowledge base for decision making but stands in contrast to the intermittent nature of clinical consultations. Health care professionals already feel overwhelmed with data, and patients may not wish to be monitored [25,26]. In addition, studies from the field of dependable computing have shown that systems produce many false-positive alerts, calling the usefulness of these applications into question [27,28]. Such issues may lead to unintended consequences that can, in turn, result in poor patient outcomes and safety risks [29-32]. Health care data are subject to particular concerns and special privacy and confidentiality protections. The use of wearable sensor devices for health care therefore introduces additional layers of complexity surrounding information privacy, data sharing, autonomy, consent, ownership, data access, and data valuation [25].

Industry reports suggest that as a system, IoT, including wearable sensor devices, is an established emerging technology, estimating 5-10 years until it reaches its “plateau of productivity” [33]. The area is, therefore, ripe for scientific investigation. Here, it is crucial to identify emerging risks and challenges in order to anticipate and mitigate them. Therefore, we aimed to explore the opportunities, gaps, and challenges of wearable sensor devices, as one component of an IoT system, in health and care settings.

## Methods

### Overview and Ethical Approval

We conducted a qualitative interview–based study with international experts and stakeholders involved in designing, developing, and implementing wearable sensor devices in health care [34]. This work was theoretically informed drawing on sociotechnical systems theory [35]. Institutional ethical approval was obtained from the University of Edinburgh Usher Research Ethics Group.

### Sampling and Recruitment

We developed a sampling frame by searching the technical, cultural, media, academic, and health care literature to capture the various perspectives and areas of expertise surrounding wearable sensor devices in health care settings. This was used as a basis for recruitment and was supplemented by consulting with local experts at the University of Edinburgh in culture, science and technology, and IoT studies. We used a purposive sampling strategy aiming for maximum variation in order to gain insights into the range of perspectives that need to be taken into account when considering the implementation and adoption of wearable sensor devices in health care settings. In so doing, we sought out experts with experience across a range of health care settings, technological functionalities, and disciplinary backgrounds [36]. This purposive sampling approach was supplemented by snowball sampling using recommendations from the initial purposive sample [37]. 

Participants were recruited by emails that were sent to publicly available email addresses; each message introduced the project and requested an interview. Interviews were scheduled with respondents who replied and provided informed consent to participate. We sent a total of 79 emails to potential participants. A follow-up email was sent to nonresponders 1 week later, after which we ceased further contact assuming no interest in participation. We stopped recruitment when we reached thematic saturation (ie, when no new themes emerged in the concurrent analysis) [38].

### Data Collection 

We collected data through in-depth, semistructured, one-to-one, qualitative telephone and video interviews from May to July 2019. Identified experts were located in various international settings. Interviews were guided by a topic guide exploring personal and professional backgrounds and experiences of, and involvement with, wearable sensor devices in health and care settings (see Textbox 1) [39]. The questions were based on the existing literature in order to understand the current opportunities, gaps, and challenges associated with the use of wearable sensor devices in health and care settings. They were tailored to the specific characteristics of participants and evolved in light of emerging findings. In doing so, we explored visions of technologies; experiences of implementing, using, and researching technologies, including benefits and risks; and features conducive to accelerating the use of wearable sensor devices in health and care settings.

**Example topic guide.**

1. Interviewee’s background: current position and role in relation to health information technology.

2. Their vision surrounding the use of wearable sensor devices in health care:

- What different models of wearable sensor devices in health care exist?
- Most promising developments to look out for and their benefits
- Devices and technologies to collect and analyze data
- Integration with clinical data and interfacing with other technologies.

3. Experiences of technological innovation in health care:

- Experiences and lessons learned (eg, consent models, data security, motivating users, scale, and data analytics)
- How does health care differ from other sectors? Anything we can learn?
- Which factors hinder developments and how might these be addressed?

4. Accelerating innovation in health care:

- Perceived risks
- How can this approach be scaled up?
- What is the role of regulation and government?

5. Anything else?

The interviews were recorded using Zoom conferencing (Zoom Video Communications) or an encrypted digital audio recorder. We read transcripts multiple times annotating with notes to immerse ourselves in the data. We also kept a field journal noting impressions and emerging analytical thoughts.

Following the interviews, the digitally recorded audio files were transferred via a secure virtual private network to a secure site for professional transcription. Interview transcripts, notes, and file names were anonymized.

### Data Analysis 

IA, a surgeon and health services researcher, led the data analysis. The analysis drew on sociotechnical systems theory, which assumes reciprocal shaping of technologies and people by their interactions with each other [40]. Through this lens, we viewed the process of integrating wearable sensor devices into health care practices as a sociotechnical practice where technologies shape human behaviors and these, in turn, shape technological design. We analyzed data concurrently with ongoing data collection and stopped collecting new data when no new themes emerged in the analysis.

Completed transcripts and field notes were organized and coded using NVivo 12 (QSR International) [41]. We used a concept-driven thematic approach based on the interview guide and key concepts in health [42], technology [43,44], unintended consequences in health information technology (HIT) [45], and innovation to analyze the data [46]. In doing so, we adhered to the six key features of an inductive and deductive coding approach, namely (1) developing the codebook with key themes a priori, (2) testing code reliability, (3) summarizing data and identifying key themes, (4) applying coding templates and new codes, (5) connecting codes and identifying themes, and (6) corroborating and legitimating coded themes [47]. We used constant comparison to examine themes, irregularities, and patterns across interviews [48].

## Results

### Overview

We conducted 16 in-depth, semistructured interviews lasting between 30 and 75 minutes with experts in technology, business, health care, and innovation (see Table 1); 1 interview was conducted in person and 15 interviews were audio recorded. All study participants had at least 5 and up to 20 years’ experience in their fields. Contributing participants were located in Europe, the United Kingdom, and the United States.

The analysis generated data around four main themes that demonstrated the significant potential of wearable sensor IoT in health care, but also highlighted issues around data provenance and quality; transforming health and care relationships; blurring of business, consumer, health, and clinical boundaries; and issues around privacy, confidentiality, and data ownership (see Textbox 2).

Participants agreed that the application of wearable sensor devices in health and care has significant potential to positively transform health outcomes. Interviewees gave examples of the potential to enhance alerting, increase patient and public involvement, facilitate processing of information, and improve efficiency, while increasing the reliability and reducing the cost of manual data capture. Participants emphasized different issues, according to their professional backgrounds. For example, academics and researchers focused on social implications; technologists and engineers on increased connectivity; and clinicians on patient safety, care processes, and quality of care.

So a lot of what I’m more focused on is wearables in everyday life, so the way in which they inspire people to adapt particular modes of care and wellness, so the idea that one wears a Fitbit as a motivational tool or a social tool to share their step counts with people as then creating another form of motivation.

Participant #3, historian, United States

By delivering care outside the hospital you save a lot more money. By keeping patients outside the hospital with these devices that can proactively monitor and maybe even prevent certain events. That gives significant cost savings to the industry.

Participant #12, engineering and business, United States

Notably, participants expressed little dissonance in conceptualizing wearable sensor devices as a component of an information ecosystem consisting of various technologies and humans. As such, technological functions were seen as vessels that collected and disseminated data that would then travel through the ecosystem and impact human and organizational behavior, as data were interpreted and acted on.

I realized that the real value in this whole equation is data, and if you can get the data and you can own the data from a wearable device, then we can turn that into meaningful value for someone with an ecosystem, whether that’s patients, providers, caregivers, sports teams, someone within the health universe.

Participant #5, business and innovation, United States

However, many of these hopes were associated with future developments, interoperability of technologies, improved fidelity to physiology, and enhanced machine learning and predictive capabilities. For the most immediate future, participants highlighted a number of concerns that were perceived to inhibit the potential of wearable sensor devices in health and care settings. These all related to the intersection of social and technological dimensions and will be discussed in turn.

**Table 1:** Study participants’ expertise, industries, and work locations.

Participant No.	Expertise	Sector	Location
1	Product developer	Technology	United States
2	Science and technology studies	Academia	United Kingdom
3	Historian	Academia	United States
4	Engineering and business	Academia Technology	United States
5	Neuroscience, psychology, and business	Technology	United States
6	Law	Academia	United States
7	Science and technology studies	Academia	Northern Europe
8	Health care and life sciences	Consulting	United Kingdom
9	Design and innovation Business	Health care and pharmaceuticals	United States
10	Social and behavioral science	Academia	Western Europe
11	Electrical, computer, and biomedical engineering	Academia	United States
12	Business and engineering	Technology	United States
13	Surgery	Health care Academia	United Kingdom
14	Biomedical engineering	Consulting (business)	United Kingdom
15	Rehabilitation Electrical engineering	Public health	United States
16	Quality and improvement Nursing	Health care	United States

**Key themes and subthemes identified in our analysis.**

Significant potential of wearable sensor devices in health and care:

1. From data to action: the role of data provenance and data quality:

- Issues of objective measurement of activity and translation into signals for effective evidence-based decision making
- Data provenance and quality as central considerations for effective adoption and diffusion
- Risk of data overload among health care professionals.

2. Transforming relationships through wearable sensor devices and associated data:

- Integration of devices and data with existing workflows of health care professionals
- Wearable sensor devices as potentially perceived mechanisms of surveillance and control
- Disruption of the traditional hierarchy of specialist information processing through health and care professionals
- Impact on the provider and patient relationship.

3. Increasing blurring of business and consumer as well as health and care boundaries:

- Tension between consumer product and use for wider public good
- Asymmetry of interests through financial sustainability of wearable sensor devices
- Need for new business models.

4. Privacy, confidentiality, and data ownership:

- Lack of regulation of data flows between health care, commercial, and private spaces
- Need for development of standards, especially for apps that were not designed primarily for use in health- and care-related settings.

### From Data to Action: The Role of Data Provenance and Data Quality

Participants highlighted that incorporating unannotated data into health and care practices was, at present, problematic, as it decoupled social and technological dimensions and thereby assumed that (1) data presented a robust and consistent measurement of activity, (2) data could be translated into recognizable signals, and (3) data were appropriately situated in context for dependable, effective, and evidence-based decision making. A key issue here was establishing baseline, “normal,” pathologic, and “unusual” not otherwise-specified activity. High hopes were placed on algorithms to support the recognition of unusual signals that could then trigger clinical intervention.

...using algorithms to interpret what normal activity for a patient is, or what a normal baseline is for that patient preoperatively. And then using algorithms and AI [artificial intelligence] to try and refine that and improve, either predicting that patients are deteriorating at an early stage; or purely for providing individual feedback to say, “actually, you’re progressing well,” or “you’re not progressing as much as we’d expect.”

Participant #13, surgery, United Kingdom

Data provenance and quality, therefore, became central considerations for effective use of data generated by wearables in health and care settings. This was particularly true where observed data patterns may not have distinct correlates to disease mechanisms or physiology. This makes it difficult to confidently base clinical decisions or interventions on them. Here, participants made distinctions between clinical, health, and well-being data. For example, variability in data used for individual empowerment was sufficient and acceptable (eg, increasing a person’s daily step count), while variability in clinical parameters (eg, the measurement of blood glucose levels that may indicate the need to give an insulin injection) was viewed as problematic, as resulting human actions had potentially impactful consequences.

...now we’ve got abundant data and we train algorithms, particularly if I do things like I drive a deep learning algorithm hard, then I’m going to find some pattern and some interesting stuff emerging about how you...you know, is that actually an artefact of the data? Yes, it’s a real pattern, but is it actually meaningful in any true physiological sense that’s a response of causal reality or only correlative?

Participant #4, engineering and innovation, United States

In addition, participants raised concerns that health care staff were already overloaded with data and that the integration of further, potentially irrelevant, data from additional devices could unnecessarily burden already-busy professionals and, therefore, fail to mobilize an important actor in the ecosystem. The interviews broadly reflected a presumption that device data would be actioned in consultation with a clinician and that the devices would serve to augment the clinician’s capability in making decisions, rather than eliminate them from the care process.

The challenge is that, as yet, data that’s collected from wearables is very difficult to integrate into the health record of a patient. So, a patient turning up at their doctor’s with a whole list of data and tracking information from their wearable isn’t necessarily going to find a welcome recipient, in terms of the doctor.

Participant #8, consulting, United Kingdom

### Transforming Relationships Through Data Generated by Wearable Sensor Devices

Integration of devices and data with existing workflows of health care professionals was viewed as crucial for wearable sensor devices to become active parts of the ecosystem, as this was seen to facilitate the exchange of information between social and technological dimensions. However, participants shared concerns about the impact of new types of data on care processes and relationships in clinical work practices. Some participants viewed the increasing focus on data as challenging the autonomy of health care professionals in making decisions about how and when to provide care and as a method of surveillance and control. This was particularly apparent in interviews with clinicians.

One of the biggest issues that we faced was with our nursing staff and their concern over sort of this idea of big brother and “people watching over what I’m doing,” and “I’m a nurse, I know how to turn a patient, I really don’t need a device to tell me how to do that.”

Participant #16, quality improvement and nursing, United States

There were also some concerns that the wide availability of data generated by wearable sensor devices to a range of actors, including device companies, family members, and caregivers, may disrupt the traditional hierarchy of specialist information processing through health care professionals. Traditionally, clinicians captured and interpreted information in context at discreet moments in the consultation, and wearable sensor devices may disrupt this information flow.

There are a lot of people who are contacting clinicians or caregivers and saying, “what’s this?” On one hand, it’s positive, but on the other hand, the health care system isn’t structured for that type of engagement. And so, I think you’re seeing a range of engagement from people around these devices.

Participant #5, business and innovation, United States

Health care providers raised concerns about becoming increasingly distanced from the patient, as they were getting health- and care-related information from sources other than the patient directly, and without recognized professional interpretation. This distance has implications on responsibility and accountability for information collected and the subsequent decisions and actions taken, or missed.

For doctors, I think their big concern is, well, is my relationship with my patient now going to be diminished ‘cause they have another place that they can go to track their symptoms and get information, so maybe I don’t become that first-line person that they want to talk to?

Participant #9, health care design and innovation, United States

### Increasing Blurring of Business and Consumer as well as Health and Clinical Boundaries

Wearable sensor devices were seen as a key example of blurring boundaries between business and consumer as well as health and care worlds, and this posed challenges associated with the assumptions underlying the design of the technology and the contexts of use. Technology and business actors saw the promotion of a commercial market for consumer technology health and care applications, aligning this unproblematically with promoting public health.

Companies like Samsung and Alexa, Apple, Google, they see health care as a potential pinpoint that their technology could potentially solve, and so it’s a good thing, I think.

Participant #5, business and innovation, United States

However, for others, aligning actors around financing and development was a major rate-limiting step for the potential wider public good, as companies came with their own commercial strategies tied to their own proprietary standards. These participants were skeptical that data generated by wearable sensor devices could be used to allow better management of individual health conditions. This indicates a misalignment of higher-level health policy goals and the reality of evidencing improved outcomes for patients and business functions using technology in health and care.

One of the upshots is it’s encouraging healthier behavior overall, I’m for that. I want people to live their healthiest best lives. And if there’s a technology that allows us to do that, I’m a little skeptical, but if there is, I’m for it.

Participant #6, law, United States

Payments and financial sustainability of wearable sensor devices in health care was raised as a concern about the technology partners’ motives, perhaps contributing to a lack of adoption in clinical contexts. In addition to creating products and services to support good health outcomes, uncertainty remained about sustainability. This included issues around maintenance and product iteration, updating equipment and devices, continued use of devices by health care professionals and patients, aligning of different interests over time, and financing of ongoing data analyses. These queries indicated a desire to rethink how goods and services are designed and delivered when technology is closely intertwined with health care. Existing business models reflected in existing technological designs were considered inappropriate.

Our business model was one which we assume we’d have to make money on subscription income, because in order to deliver a good experience, the bill or materials costs can be high, and to make the economics stack up, you couldn’t do it by charging somebody up front for the device. The price would be too high, it could be a couple thousand dollars at that point.

Participant #4, engineering and innovation, United States

### Privacy, Confidentiality, and Data Ownership

Participants also raised questions about misuse of data, balancing how much data to collect, data ownership, data portability, and implications for insurability. It was unclear how the data generated in devices were regulated, and many participants raised concerns of data flows between health care, commercial, and private spaces. The overarching concern of clinicians in this area was the impact of altered data flows on trust, given the lack of clarity over control, sharing, and repurposing of health-related data.

I think the big question [that] will come about is actually, well, who owns that data? And who uses that data? Because the problem is, if you then have it on a Google server, then is it not only accessible by the researchers and by the clinicians but accessible by the company themselves. Then it only takes, kind of, one example of that industry partner using that data for something else, that potentially patients haven’t consented for, that it then starts to become quite a big issue and then trust, kind of, is degraded...

Participant #13, surgery, United Kingdom

Important considerations for developers and implementers included the development of technology standards, especially as many applications were not designed primarily for use in health- and care-related settings. This was perceived to promote trust among the user community and ensure a closer fit between technological design and clinical practice.

What is needed also are some standards. So a lot of these technologies, particularly in the consumer technologies, have been developed as a consumer product and they won’t necessarily conform to the health care standards that are needed, either in terms of the technology, the interoperability standards, in terms of the health care side of it, they won’t have passed as a medical device for health care.

Participant #8, consulting, United Kingdom

Similarly, participants also called for increasing regulatory efforts to ensure that devices that were applied to health and care systems achieved maximum benefit, while minimizing harm.

It’s to work collaboratively with regulators so that we expedite and maximize the potential of the technologies that we have without doing any harm to patients.

Participant #14, biomedical engineering and consulting, United States

## Discussion

### Principal Findings

Wearable sensor devices have significant potential, particularly in relation to promoting patient engagement. However, achieving these potential benefits is dependent on addressing the current gap between data created and meaningful interpretation in health and care contexts. Current applications cannot fulfill their potential if they do not yield benefits for clinical users and thereby integrate effectively within the existing ecosystem of social and organizational actors. This may be achieved through modifying technological design to allow data to integrate effectively into health care professional decision making, negotiating of blurring lines between consumer and medical care, and appropriate regulatory contexts incorporating pervasive monitoring of health.

### Strengths and Limitations

We have drawn on a range of perspectives from various sectors to explore the potential benefits and challenges of incorporating data generated by wearable sensor devices into health and care settings. The breadth of participant expertise across disciplines, projects, and national contexts allowed triangulation and constant comparison of generated data. These in-depth interviews have identified a range of barriers that stakeholders need to negotiate and provide an overview of the extended health care ecosystem of care providers and technology developers that can help to maximize the potential benefits of this technology. Actively anticipating and mitigating risks, before they impact negatively on the safety and quality of health and care, can help to inform future system design, implementation, and optimization.

Some limitations of this study include the diversity of applications and contexts discussed, so that it was at times difficult to extract common themes, and a potential lack of generalizability of findings across settings with different regulatory environments (as most participants came from the United States). Providing accessible and broad coverage of key concerns in the field of applying wearable sensors in health care may also have compromised in-depth insights into the concerns of different stakeholder groups. Unfortunately, none of the insurance companies that we reached out to on multiple occasions responded to our invitation to be interviewed, so we were not able to gain insights into these perspectives. There may also have been pressure on participants to construct knowledge during the interviews. For example, participants responded to a handful of the interview questions with “good question,” and their responses likely represented efforts to collate their thoughts and experiences into durable representations of the subject [49].

### Integration of Findings With the Current Literature

The value of this work lies in providing accessible and broad coverage of key concerns in the field of applying wearable sensors in health and care settings. It shows that wearable sensor devices should not be viewed as stand-alone technologies in isolation, but as part of an emerging IoT ecosystem of actors. In doing so, they emerge as a key driver of service redesign in health care innovation. Our analysis revealed tensions in overlapping goals and agendas that challenge the effective integration of data generated by wearable sensor devices into health and care settings. This finding is similar to other sociotechnical studies of HIT where a range of actors with different agendas have to align for successful adoption [50-52].

Wearable sensor devices and associated data, however, do add an extra layer of complexity. Data fluidity means that many stakeholders have to be mobilized in different ways to ensure that data are captured and relayed meaningfully to various actors. In addition, this case also vividly illustrates tensions between visions and real-life challenges of using mobile devices to connect health-related data to meet public health and consumer market agendas. It further highlights the need to develop new business models that do not exploit certain groups of stakeholders for commercial agendas.

Data generated by wearable sensor devices also create new challenges and opportunities for health care professional work practices and relationships with patients. Previous work has surfaced unintended consequences of HIT on communication, coordination of care, and clinical work practices that our findings confirm [53]. In addition, we have shown how data from wearable sensor devices can introduce new insights for, but also disruptions to, communication and work practices of health and care professionals [54].

Given the potential consequences of acting on data improperly, or of acting on improper data, this study highlights the importance of addressing data provenance and quality and of presenting relevant information. Existing commercial stakeholders are increasingly building on this. Health data aggregation companies, such as 1upHealth [55], Open mHealth [56], Validic [57], Seqster [58], Human API [59], and Apple combine information from many data sources, wearables, apps, sensors, and electronic health records, producing information for health care organizations, clinicians, and users. New start-ups like Conversa and LifeWIRE have added human and nonhuman intermediaries to help patients and providers navigate aggregated health data [60,61]. Generally, efforts promoting solutions that blur lines between consumer and medical care too much are unlikely to be effective, as a functioning ecosystem requires clinicians to be active actors to realize the potential benefits of wearable sensor devices in health and care settings. Nevertheless, changing social contexts, as seen in the COVID-19 pandemic, may change these dynamics. For example, companies like Current Health have expanded their range of sensors and connections to peripheral devices providing patient health data for remote monitoring [62].

Overall, while there is, at present, limited interest in bringing data generated by wearable sensor devices into the care consultation from the provider side [63-65], the ongoing COVID-19 pandemic has mandated a reconsideration for how best to provide care at a distance using wearable sensor devices. Even within this unexpected contextual shift, interconnected devices and data will need to represent the communications, contexts, conversations, and social connectedness of care interactions in order to maximize usefulness and effectiveness for a range of stakeholders [66-68].

### Policy Recommendations and Implications for Practice Emerging From This Work

Wearable sensor devices are best conceptualized as data-generating components within a distributed information system. They are not a simple device implementation; rather, such devices are key components of regular input into an IoT platform in a health care ecosystem. Further mixed methods research now needs to demonstrate effects of these devices on cost and health care outcomes, how data are used, and how health care knowledge and practices are presented and represented in the data obtained.

Implications for accelerating the integration of data generated by wearables into health care practices include negotiating the fit with existing health care professional and patient relationships, mitigating adverse unintended consequences, and aligning interrelated agendas. Triangulation of the agendas of providers and those that organize care will be key, as this seems to be crucial for promoting effective self-management. Conceptualizing specific solutions as complex public health interventions with aligned business models could help to achieve this, facilitated by rigorous evaluation to achieve mainstream utility and use [69,70].

Interviewees highlighted that regulatory guidance was lacking in the field; there were also international differences in regulatory contexts. Regulators do not classify most wearable sensors as medical devices, despite increasingly blurring lines [71,72]. There is evidence to support that regulation can both facilitate and stifle innovation [73,74]. The regulatory landscape for wearable sensor devices in health care is challenging because of the distributed stakeholder network, the involvement of technical specialists, tight regulations, and privacy and security concerns. Whether data generated by wearable sensor devices will contribute to the transformation of health and care will, in part, depend on deliberate and consistent regulatory policy to reduce uncertainty around investments [75].

In the United States, such efforts are likely to be most effectively led by national bodies to facilitate widespread diffusion and sustain wearable sensors in routine practices. The United States Centers for Medicaid Services proposed rules to take effect in 2021 that clarify that devices supporting remote physiological monitoring must be defined as medical devices by the United States Food and Drug Administration and must be “reliable,” “valid,” and collect data “electronically” to qualify [76]. This proposal follows expansion of physician reimbursement fees for remote physiological monitoring in 2020. These fee schedules create a favorable environment for expansion of existing and emerging digital health services using devices to take physiological and biometric measurements [77].

### Conclusions

Our work suggests that meaningful use of wearable sensor devices in health and care settings occurs through a platform of interconnected devices and data users around a specific use case. This platform represents the sum of knowledge, practices, and contexts of health care, oriented to improve a system of care, rather than a singular focus on a specific device. Engaging the range of relevant stakeholders who participate in design and development of systems is therefore essential to maximize scale, impact, and adoption.

Wearable sensor devices have great potential in improving patient engagement and thereby contributing to preventive, diagnostic, and treatment approaches. Such technologies are, however, most likely to be successful in achieving this potential if systems can align business, professional, personal, and health systems agendas.

## Abbreviations

AI: artificial intelligence
HIT: health information technology
IoT: Internet of Things

## References

[1] Lapão LV (2016). The future impact of healthcare services digitalization on health workforce: The increasing role of medical informatics. Stud Health Technol Inform.

[2] Dimitrov DV (2016). Medical Internet of Things and big data in healthcare. Healthc Inform Res.

[3] Marques R, Gregório J, Mira Da Silva M, Lapão LV (2016). The promise of the Internet of Things in healthcare: How hard is it to keep?. Stud Health Technol Inform.

[4] Metcalf D, Milliard ST, Gomez M, Schwartz M (2016). Wearables and the Internet of Things for health: Wearable, interconnected devices promise more efficient and comprehensive health care. IEEE Pulse.

[5] Cahill J, Portales R, McLoughin S, Nagan N, Henrichs B, Wetherall S (2019). IoT/sensor-based infrastructures promoting a sense of home, independent living, comfort and wellness. Sensors (Basel).

[6] Piwek L, Ellis DA, Andrews S, Joinson A (2016). The rise of consumer health wearables: Promises and barriers. PLoS Med.

[7] (2020). Medtech and the Internet of Medical Things. Deloitte.

[8] Thümmler C, Fricker SA, Thümmler C, Gavras A (2015). Digital health. Requirements Engineering for Digital Health.

[9] Belliger A, Krieger DJ, North K, Maier R, Haas O (2018). The digital transformation of healthcare. Knowledge Management in Digital Change: New Findings and Practical Cases.

[10] Hung K, Zhang Y, Tai B (2004). Wearable medical devices for tele-home healthcare. Proceedings of the 26th Annual International Conference of the IEEE Engineering in Medicine and Biology Society.

[11] Price L (2020). The digital health hype cycle 2019. Healthcare.Digital.

[12] Biesdorf S, Niedermann F (2014). Healthcare's digital future. https://www.mckinsey.com/industries/healthcare-systems-and-services/our-insights/healthcares-digital-future.

[13] McKinsey Global Institute (2017). The Productivity Puzzle: A Closer Look at the United States.

[14] Qureshi F, Krishnan S (2018). Wearable hardware design for the Internet of Medical Things (IoMT). Sensors (Basel).

[15] Athavale Y, Krishnan S (2018). A device-independent efficient actigraphy signal-encoding system for applications in monitoring daily human activities and health. Sensors (Basel).

[16] Rathore MM, Ahmad A, Paul A, Wan J, Zhang D (2016). Real-time medical emergency response system: Exploiting IoT and big data for public health. J Med Syst.

[17] Bhide A, Muthukumar S, Prasad S (2018). CLASP (Continuous lifestyle awareness through sweat platform): A novel sensor for simultaneous detection of alcohol and glucose from passive perspired sweat. Biosens Bioelectron.

[18] Istepanian R, Hu S, Philip N, Sungoor A (2011). The potential of Internet of m-health Things. Proceedings of the Annual International Conference of the IEEE Engineering in Medicine and Biology Society.

[19] Kario K, Tomitani N, Kanegae H, Yasui N, Nishizawa M, Fujiwara T, Shigezumi T, Nagai R, Harada H (2017). Development of a new ICT-based multisensor blood pressure monitoring system for use in hemodynamic biomarker-initiated anticipation medicine for cardiovascular disease: The national IMPACT program project. Prog Cardiovasc Dis.

[20] Fagherazzi G, El Fatouhi D, Bellicha A, El Gareh A, Affret A, Dow C, Delrieu L, Vegreville M, Normand A, Oppert J, Severi G (2017). An international study on the determinants of poor sleep amongst 15,000 users of connected devices. J Med Internet Res.

[21] Kim J, Ryu B, Cho S, Heo E, Kim Y, Lee J, Jung SY, Yoo S (2019). Impact of personal health records and wearables on health outcomes and patient response: Three-arm randomized controlled trial. JMIR Mhealth Uhealth.

[22] Hardcastle SJ, Hince D, Jiménez-Castuera R, Boyle T, Cavalheri V, Makin G, Tan P, Salfinger S, Tan J, Mohan GR, Levitt M, Cohen PA, Saunders C, Platell C (2019). Promoting physical activity in regional and remote cancer survivors (PPARCS) using wearables and health coaching: Randomised controlled trial protocol. BMJ Open.

[23] Wang JB, Cadmus-Bertram LA, Natarajan L, White MM, Madanat H, Nichols JF, Ayala GX, Pierce JP (2015). Wearable sensor/device (Fitbit One) and SMS text-messaging prompts to increase physical activity in overweight and obese adults: A randomized controlled trial. Telemed J E Health.

[24] Vogels EA (2020). About one-in-five Americans use a smart watch or fitness tracker. Pew Research Center.

[25] Mittelstadt B (2017). Ethics of the health-related Internet of Things: A narrative review. Ethics Inf Technol.

[26] Hall A, Walton G (2004). Information overload within the health care system: A literature review. Health Info Libr J.

[27] Dewsbury G, Clarke K, Rouncefield M, Sommerville I (2002). Designing appropriate assistive technology for home users: Developing dependable networks. Proceedings of the Inclusive Design and Mobility Response in Indoor/Outdoor Public Buildings and Facilities: CIB Working Group W084 – Building Non-Handicapping Environments.

[28] Sommerville I, Dewsbury G (2007). Dependable domestic systems design: A socio-technical approach. Interact Comput.

[29] Zheng K, Abraham J, Novak LL, Reynolds TL, Gettinger A (2018). A survey of the literature on unintended consequences associated with health information technology: 2014–2015. Yearb Med Inform.

[30] Alami H, Gagnon M, Fortin J (2019). Some multidimensional unintended consequences of telehealth utilization: A multi-project evaluation synthesis. Int J Health Policy Manag.

[31] Aarts J, Ash J, Berg M (2007). Extending the understanding of computerized physician order entry: Implications for professional collaboration, workflow and quality of care. Int J Med Inform.

[32] Ash JS (2003). Some unintended consequences of information technology in health care: The nature of patient care information system-related errors. J Am Med Inform Assoc.

[33] Panetta K (2018). 5 trends emerge in the Gartner hype cycle for emerging technologies. Gartner.

[34] Crowe S, Cresswell K, Robertson A, Huby G, Avery A, Sheikh A (2011). The case study approach. BMC Med Res Methodol.

[35] Braithwaite J, Runciman WB, Merry AF (2009). Towards safer, better healthcare: Harnessing the natural properties of complex sociotechnical systems. Qual Saf Health Care.

[36] Creswell JW (2007). Qualitative Inquiry and Research Design: Choosing Among Five Approaches. 2nd edition.

[37] Biernacki P, Waldorf D (2016). Snowball sampling: Problems and techniques of chain referral sampling. Sociol Methods Res.

[38] Yazan B (2015). Three approaches to case study methods in education: Yin, Merriam, and Stake. Qual Rep.

[39] Seale C, Gobo G, Gubrium JF, Silverman D (2004). Qualitative Research Practice.

[40] Bolstrom RP, Heinen JS (1977). MIS problems and failures: A social-technical perspective, Part I: The causes. MIS Q.

[41] (2020). Download NVivo. QSR International.

[42] Braun V, Clarke V (2006). Using thematic analysis in psychology. Qual Res Psychol.

[43] Jennex M (2009). Re-visiting the knowledge pyramid. Proceedings of the 42nd Hawaii International Conference on System Sciences.

[44] Weinberger D (2010). The problem with the data-information-knowledge-wisdom hierarchy. Harvard Business Review.

[45] Schukat M, McCaldin D, Wang K, Schreier G, Lovell N, Marschollek M, Redmond S (2018). Unintended consequences of wearable sensor use in healthcare. Yearb Med Inform.

[46] Christensen CM, Bower JL (1996). Customer power, strategic investment, and the failure of leading firms. Strateg Manage J.

[47] Fereday J, Muir-Cochrane E (2016). Demonstrating rigor using thematic analysis: A hybrid approach of inductive and deductive coding and theme development. Int J Qual Methods.

[48] Padgett DK (2012). Getting started: Study design and sampling. Qualitative and Mixed Methods in Public Health.

[49] Myers MD, Newman M (2007). The qualitative interview in IS research: Examining the craft. Inf Organ.

[50] Hanseth O, Jacucci E, Grisot M, Aanestad M (2006). Reflexive standardization: Side effects and complexity in standard making. MIS Q.

[51] Robert G, Greenhalgh T, MacFarlane F, Peacock R (2010). Adopting and assimilating new non-pharmaceutical technologies into health care: A systematic review. J Health Serv Res Policy.

[52] Reeves B, Nass C (1996). The Media Equation: How People Treat Computers, Television, and New Media Like Real People and Places.

[53] Wright ER, Pescosolido B, Martin J, McLeod J, Rogers A (2011). Enter health information technology: Expanding theories of the doctor–patient relationship for the twenty-first century health care delivery system. Handbook of the Sociology of Health, Illness, and Healing.

[54] Georgiou A, Prgomet M, Lymer S, Hordern A, Ridley L, Westbrook J (2017). The impact of a health IT changeover on medical imaging department work processes and turnaround times. Appl Clin Inform.

[55] 1upHealth.

[56] Open mHealth.

[57] Validic.

[58] Seqster.

[59] Human API.

[60] LifeWIRE.

[61] Conversa.

[62] Current Health.

[63] Eramo L (2017). Do doctors care about your wearable data?. Philips.

[64] Notte C, Skolnik N (2018). Where to go with wearables. MDedge.

[65] Lim D (2019). HIMSS19: Doctors don't know what to do with data from wearables. MedTech Dive.

[66] Coiera E (2000). When conversation is better than computation. J Am Med Inform Assoc.

[67] Verghese A, Shah NH, Harrington RA (2018). What this computer needs is a physician: Humanism and artificial intelligence. JAMA.

[68] Israni ST, Verghese A (2019). Humanizing artificial intelligence. JAMA.

[69] Wight D, Wimbush E, Jepson R, Doi L (2016). Six steps in quality intervention development (6SQuID). J Epidemiol Community Health.

[70] Greenhalgh T, Wherton J, Papoutsi C, Lynch J, Hughes G, A'Court C, Hinder S, Fahy N, Procter R, Shaw S (2017). Beyond adoption: A new framework for theorizing and evaluating nonadoption, abandonment, and challenges to the scale-up, spread, and sustainability of health and care technologies. J Med Internet Res.

[71] Hwang J, Christensen CM (2008). Disruptive innovation in health care delivery: A framework for business-model innovation. Health Aff (Millwood).

[72] (2019). Device software functions including mobile medical applications. US Food and Drug Administration.

[73] (2014). Medical devices: Software applications (apps). Medicines and Healthcare products Regulatory Agency.

[74] Gann DM, Wang Y, Hawkins R (2010). Do regulations encourage innovation? The case of energy efficiency in housing. Build Res Innov.

[75] te Kulve H, Boon W, Konrad KT, Schuitmaker TJ (2018). Influencing the direction of innovation processes: The shadow of authorities in demand articulation. Sci Public Policy.

[76] Stewart LA (2011). The impact of regulation on innovation in the United States: A cross-industry literature review. https://itif.org/publications/2011/11/14/impact-regulation-innovation-united-states-cross-industry-literature-review.

[77] (2020). Proposed policy, payment, and quality provisions changes to the Medicare physician fee schedule for calendar year 2021. https://www.cms.gov/newsroom/fact-sheets/proposed-policy-payment-and-quality-provisions-changes-medicare-physician-fee-schedule-calendar-year-4.

